# Uterine Trauma and Intrauterine Fetal Death Caused by Seatbelt Injury

**DOI:** 10.1155/2019/5262349

**Published:** 2019-11-16

**Authors:** Junsuke Muraoka, Teruo Otsuka, Aya Yamauchi, Kiminari Terao

**Affiliations:** Department of Obstetrics and Gynecology, Perinatal Medicine, Miyazaki Prefectural Nobeoka Hospital, 2-1-10 Shinkoji, Nobeoka, Miyazaki 882-0835, Japan

## Abstract

We present a case of uterine trauma and intrauterine fetal death caused by seatbelt injury. A 37-year-old primigravida at gestational week 24 was involved in a single-car accident when traveling as a front-seat passenger and wearing a three-point seatbelt. Fetal heart rate monitoring patterns revealed fetal demise, and computed tomography revealed intraperitoneal bleeding due to damage to the uterine vessels and placental lacerations across the seatbelt-compressed region. Intensive treatment, including transfusion therapy and surgical laparotomy, prevented the loss of her life but not that of the fetus. Seatbelt use can reduce the overall mortality associated with motor vehicle crashes. Pregnant women must be educated on the proper use of restraints while traveling in motor vehicles.

## 1. Introduction

Motor vehicle crashes account for >50% of all traumas incurred during pregnancy, with 82% of fetal deaths occurring during those crashes [1]. Organ damage, fracture, and hemorrhage can cause maternal and fetal morbidity and mortality. Wearing a properly adjusted and positioned seatbelt in a motor vehicle during pregnancy is effective at reducing the risk of adverse pregnancy outcomes [2–4]. Here, we describe an educational case of uterine trauma and intrauterine fetal death caused by an incorrectly positioned seatbelt; we also present characteristic images of uterine bleeding and placental lacerations.

## 2. Case Presentation

**Figure 1:**
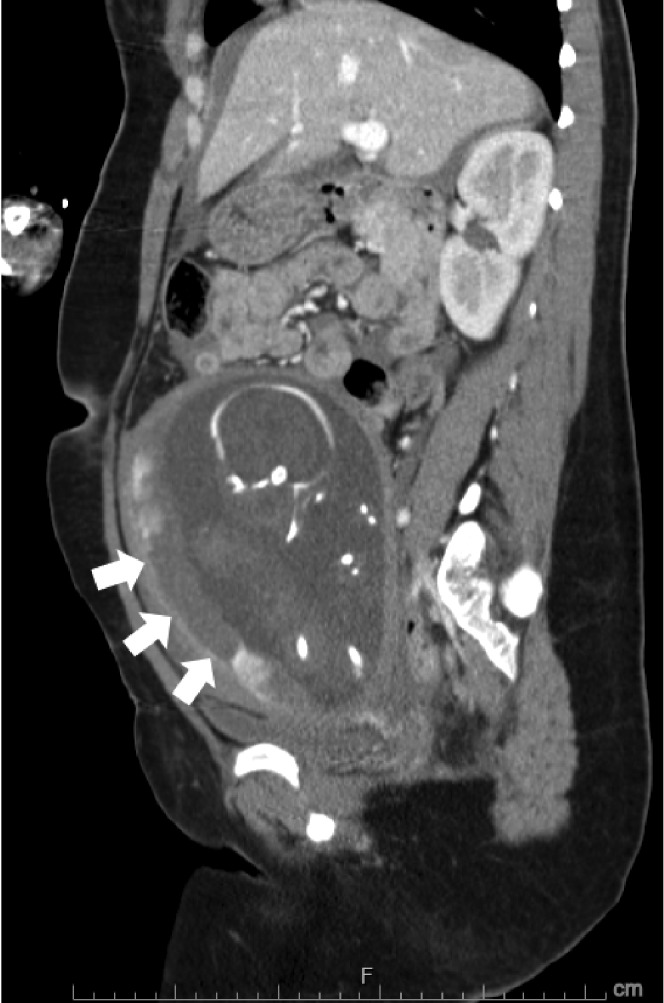
Computed tomography of the abdominopelvic region shows placental damage over the seatbelt-compressed area with a well-circumscribed low-density area (white arrows).

**Figure 2:**
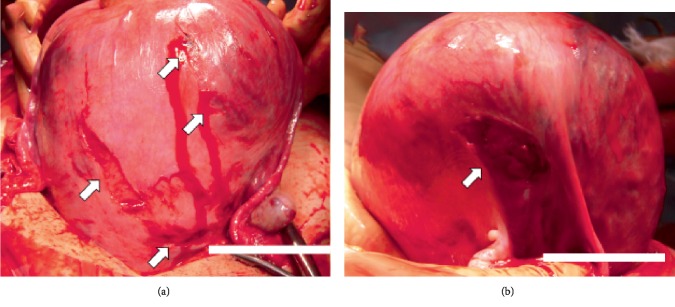
White arrows indicate uterine bleeding of the anterior surface (a) and right-side of wounds (b) during the operation prior to uterine incision.

**Figure 3:**
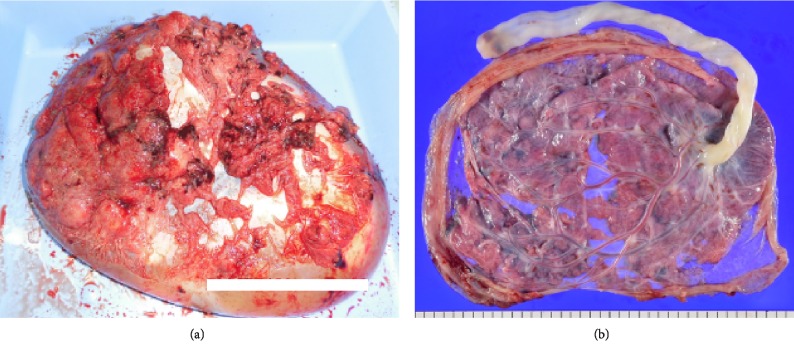
Findings of the placenta on the maternal aspect just after en caul delivery (a). The fetal aspect (b) indicating placental lacerations.

A 37-year-old primigravida at 24 + 0/7 gestational weeks was involved a single-car accident at a speed of approximately 50 km/h. She was traveling in the left front passenger seat, had worn a three-point seatbelt restraint, and had the supplemental restraints and airbag system deployed. She was transported to our hospital by ambulance 1 h after the event. On examination, her vital signs were as follows: Glasgow Coma Scale, E4V5M6; blood pressure level, 90/50 mmHg; and heart rate, 90 beats/min. She suffered seatbelt injuries to her right thorax and entire lower abdomen and presented such severe pain to her lower abdomen that she was unable to move her body. Fetal heart rate monitoring indicated bradycardia of 80–90 beats/min with loss of variability that led to rapid demise. Whole-body computed tomography revealed intraperitoneal bleeding due to vessel injury around the uterus and placental lacerations over the seatbelt-compressed region (Figure 1). Blood tests revealed D-dimer coagulopathy of 31.7 *µ*g/mL (normal level, <1 *µ*g/mL) and fibrinogen degradation product of 58.0 *µ*g/mL (normal level, <10 *µ*g/mL). Rapid and massive blood transfusion therapy, insertion of an intra-aortic balloon occlusion catheter, and emergency laparotomy under the stabilization of maternal hemodynamics were immediately performed. Bleeding occurred all across the uterine surface during the operation (Figure 2). After delivery of a deceased male baby weighing 640 g via uterine incision, the extensive bleeding surrounding the uterine vessels was stopped using sutures. The total intraoperative hemorrhage was 2500 mL, and total 12 units of red blood cells, 12 units of fresh frozen plasma, and 20 units of platelets were transfused. The mother was admitted to the intensive care unit and discharged after 2 weeks of hospitalization. The placenta was macroscopically lacerated into several parts (Figure 3), and ischemic changes were microscopically noted. Written informed consent was obtained from the patient prior to publication of this case report and the accompanying images.

## 3. Discussion

Obstetric complications occurring due to trauma include placental abruption, uterine rupture, preterm labor, and direct fetal injury [5]. Examination techniques are available for the assessment of both maternal and fetal conditions following motor vehicle injuries. In urgent situations, computed tomography is helpful for the rapid evaluation of maternal general conditions. In our patient, a notable low-density area of the placenta indicated placental laceration along the seatbelt-compressed area. This condition was clinically similar to placental abruption and led to subsequent intrauterine fetal death. In addition to this placental damage, numerous hemorrhages from the uterine surface were found. Bleeding from the right lateral aspect of the uterus persisted despite uterine involution after cesarean delivery. We speculated that the seatbelt caused the uterine hemorrhages and placental lacerations.

Motor vehicle crashes during pregnancy require rapid clinical judgment to assess the extent of the maternal and fetal damages. The indication for urgent surgical treatment would depend on maternal circulatory condition, blood coagulability, gestational age, and fetal well-being assessment. In cases of maternal cardiac arrest, perimortem cesarean section should be considered early in the resuscitation of the trauma victim when fetal viability is a concern and the pregnancy has extended beyond 23–24 weeks of gestation [6]. Emptying the uterus may improve maternal cardiac output and increase the likelihood of successful maternal resuscitation [7]. In the present case, the fetal heart rate patterns on admission implied an impending fetal demise and the mother remained in a critical but stable condition under transfusion therapy. Therefore, emergency laparotomy was performed after systemic workup of the source of bleeding and other organ damage. Because uterine injuries with coagulopathy were obvious from the operative findings, cesarean section was more favorable for delivery of the deceased neonate than labor induction was.

The use of three-point seatbelt restraints during pregnancy is highly recommended to reduce both maternal and fetal morbidity and mortality [8]. When women were properly restrained, adverse fetal outcomes occurred in 29% of motor vehicle crashes, whereas in women who were improperly restrained, adverse fetal outcomes occurred in 50% of motor vehicle crashes [2]. Early in mid-gestation, the pregnant abdomen may not be large enough to place the lap seatbelt below the abdominal dome. As in the present case, placement of the lap seatbelt over the dome of the uterus increases pressure transmission and uterine injury risk during a significant crash. Therefore, pregnant women should appropriately position seatbelts throughout gestation.

In summary, appropriate and prompt evaluation of maternal and fetal injuries is essential for subsequent intervention in cases of blunt trauma during pregnancy. Pregnant women should be educated on the proper use of seatbelt restraints while traveling in motor vehicles.
